# Neighbourhood green space, physical function and participation in physical activities among elderly men: the Caerphilly Prospective study

**DOI:** 10.1186/1479-5868-11-40

**Published:** 2014-03-19

**Authors:** Yi Gong, John Gallacher, Stephen Palmer, David Fone

**Affiliations:** 1 Sustainable Places Research Institute, Cardiff University, Cardiff, Wales, UK; 2 Institute of Primary Care & Public Health, School of Medicine, Cardiff University, Cardiff, Wales, UK

**Keywords:** Green space, Lower extremity physical function, Physical activity, Elderly men

## Abstract

**Background:**

The built environment in which older people live plays an important role in promoting or inhibiting physical activity. Most work on this complex relationship between physical activity and the environment has excluded people with reduced physical function or ignored the difference between groups with different levels of physical function. This study aims to explore the role of neighbourhood green space in determining levels of participation in physical activity among elderly men with different levels of lower extremity physical function.

**Method:**

Using data collected from the Caerphilly Prospective Study (CaPS) and green space data collected from high resolution Landmap true colour aerial photography, we first investigated the effect of the quantity of neighbourhood green space and the variation in neighbourhood vegetation on participation in physical activity for 1,010 men aged 66 and over in Caerphilly county borough, Wales, UK. Second, we explored whether neighbourhood green space affects groups with different levels of lower extremity physical function in different ways.

**Results:**

Increasing percentage of green space within a 400 meters radius buffer around the home was significantly associated with more participation in physical activity after adjusting for lower extremity physical function, psychological distress, general health, car ownership, age group, marital status, social class, education level and other environmental factors (OR = 1.21, 95% CI 1.05, 1.41). A statistically significant interaction between the variation in neighbourhood vegetation and lower extremity physical function was observed (OR = 1.92, 95% CI 1.12, 3.28).

**Conclusion:**

Elderly men living in neighbourhoods with more green space have higher levels of participation in regular physical activity. The association between variation in neighbourhood vegetation and regular physical activity varied according to lower extremity physical function. Subjects reporting poor lower extremity physical function living in neighbourhoods with more homogeneous vegetation (i.e. low variation) were more likely to participate in regular physical activity than those living in neighbourhoods with less homogeneous vegetation (i.e. high variation). Good lower extremity physical function reduced the adverse effect of high variation vegetation on participation in regular physical activity. This provides a basis for the future development of novel interventions that aim to increase levels of physical activity in later life, and has implications for planning policy to design, preserve, facilitate and encourage the use of green space near home.

## Background

Physical activities provide an important way for older people to keep healthy. They have a positive effect on health and mortality [1], personal well-being, life satisfaction [2], quality of life [3,4] and preventing disabilities [5]. It is widely documented that the environment in which older people live plays an important role in promoting or inhibiting physical activity [6-8]. In particular, green space has been recognized as an important behaviour setting for physical activity [9]. The presence of a large amount of green space with good access within walking distance was found to be associated with more physical activity in the form of walking [9-13]. More recent studies show that among older adults neighbourhood green space has positive associations with physical activity in a wide range of forms including sport, gardening, walking and cycling in the US [14], the Netherlands [15], Japan [16], Chile [17] and Colombia [18].

However, it is notable that most work on this complex relationship between physical activity and neighbourhood green space has not taken into account the difference between groups with different levels of physical function, especially among older populations. A certain level of physical functional capacity is required to participate in physical activity [1,19,20]. As people get older, physical functional capacity declines and people experience a shrinking of their activity spaces and participation in physical activity [21,22]. In other words, older adults tend to rely more heavily on their local environment for day-to-day activities [23]. Older people with poorer health are found to participate in less physical activity [24-27]. Walking, as an example, is the most common form of physical activity among adults [28]. But difficulty in walking is commonly observed within older populations. The US National Health and Nutrition Examination Survey (1999-2002) reported that 21% of Americans aged 60-69 reported difficulty or inability to walk 400 meters, and this proportion increased to 30% and 49% in the 70-79 year and the 80 years and over age groups respectively [29]. Older adults from the US and Canada who have no difficulties in walking half a mile or climbing stairs reported travelling greater distances and completing more errands than those with difficulties [30].

To our knowledge, only three studies (all set in the US) have explored the role of the built environment in the relationship between lower body physical function and physical activity among the elderly [31-33]. All three studies found that the impact of the built environment on physical activity varied between older people with different levels of physical function in walking. Two studies suggested that more walkable neighbourhoods were associated with more physical activity among those with reduced lower extremity physical function [31,32]. First, a cross-sectional survey of 326 adults aged 60 years and older reported that the neighbourhood destinations (average of the shortest travel time to retail and public services) and neighbourhood design (accessibility to services, street connectivity, the condition of pavements and surroundings, pedestrian/ traffic safety and crime safety) explained more variance in neighbourhood walking among those older adults with reduced physical function than those without limitations [31]. Second, a longitudinal study of 719 adults aged 66 years and older from two US regions found that older adults with the worst lower extremity function and living in more walkable neighbourhoods (measured by street connectivity, diversity of destinations/land use) reported similar levels of active transport (e.g. walking and cycling activity) to those with better lower extremity function and living in less walkable neighbourhoods [32]. In contrast, the third study suggested the environmental effects were stronger on those with good physical function by examining 884 people aged 65 and over from five US counties. In this cross-sectional analysis, living in a more compacted area with shorter median block length was associated with more walking only among those elderly people with good lower-body strength [33]. Although these three studies provided some evidence that environment may influence different sub-groups (e.g. by lower extremity physical function) differently in physical activity, there is a gap in knowledge on the effect of the environment on physical activity for older adults with different levels of physical function.

The purpose of this study was to evaluate the association between neighbourhood green space and physical activity participation among elderly men with different levels of lower extremity physical function. We first investigated the effect of the amount of neighbourhood green space and variation in vegetation on physical activity participation, using data collected from the Caerphilly Prospective Study (CaPS) and objectively measured green space data from high resolution aerial photography. Second, we explored the effects of green space on physical activity participation by different levels of lower extremity physical function.

## Methods

The Caerphilly Prospective Study was established to study cardiovascular disease in adult men, following a general population sample of men (2,512) from Caerphilly county borough, Wales, UK aged 45-69 at recruitment in 1979 [34,35]. Subsequently, various parameters of health in older adults have been included. At the fifth phase (2002-2004), ethical approval was obtained from the South Wales Research Ethics Committee, and each subject signed their agreement to be involved. 1,225 took part in the survey. Of those, 1,036 (85%) still lived within the study area and reported a valid postcode. 1,010 (82%) reported the frequency of physical activity. These data are used in this analysis.

### Socio-demographic variables

Social class was assessed using the British Registrar General’s classification [36] and considered as a two-level factor based on manual and non-manual occupations. Education was recorded as the highest achieved qualification and classified into two groups: (1) no qualifications, (2) CSE (Certificate of Secondary Education qualifications) and above, which is equivalent to education up to age 16 years and over. Marital status (married, single/ windowed/divorced/separated) and car ownership (one or more cars, no car) were both modelled as two-level factors.

### Health status

General health status was measured by asking participants to rate their health in two questions (1) in general and (2) when compared with someone of their age using a 5-point Likert scale coded as 1: “excellent”, 2: “good”, 3: “fair”, 4: “poor”, 5: “very poor”. The internal consistency of the scale was high at 0.91. Mental health was measured using the General Health Questionnaire (GHQ-30) and a cut-off score of >=5 was used as the indicator of psychological distress. Lower extremity physical function was measured using nine items assessing the difficulty of lower extremity strength and balance [37,38]. Respondents were asked “Do you currently have difficulty carrying out any of the following activities on your own as a result of a long-term health or medical problem, or due to old age?” The activities included going up or down stairs, keeping balance, bending down, straightening up, going out the house, and walking 400 yards. They also reported whether or not they had shortness of breath when walking up hills, and when walking with others at the same age, or a fall within the last 12 months. The *α* reliability was 0.85.

### Participation in physical activities

The participants reported the frequency of their participation in each of 22 activities using a five-point response scale, where response options for each activity were coded as 1: “never”, 2: “less than monthly”, 3: “monthly”, 4: “weekly”, and 5: “three or more times a week”. The factor analysis of these 22 activities led to the identification of six factors which had an eigen value greater than one. Varimax rotation with Kaiser normalisation was used to maximise the variance of the factor loading with values greater than 0.5 used as the cut-point. Three of those activities were interpreted as one factor “physical activity”, which covers participation in (1) gardening and DIY (Do-It-Yourself), (2) visiting the coast, rivers, parks and countryside and (3) doing physical exercise including ball games, golf, jogging, walking, and bowls. Respondents who reported participating in any of three activities three or more times a week were characterized as regular participants in physical activity.

### Neighbourhood

In this study, the neighbourhood was defined as a 400 meter (a quarter-mile) radial buffer around an individual’s home, where a person could walk at a 3 mph pace for about five minutes. This definition has been widely applied in other studies on older adults [33,39,40] and is consistent with a recent qualitative study showing that the perceived walkable neighbourhood for older adults (aged 65 years or over) in the UK is around 400 meters [41]. We used two different measures to capture the characteristics of the neighbourhood green space: the quantity of green space and the variation in neighbourhood vegetation.

The quantity and variation of green space are based on the Normalized Difference Vegetation Index (NDVI). NDVI is an indicator of relative biomass and greenness, which shows the presence and condition of green vegetation [42]. It is calculated by measuring the difference between two different spectral reflectances: red and near-infrared (Equation 1). It has a value range between −1 and +1. Negative values of NDVI indicate water. Values below 0.1 but above 0 correspond to barren areas of rock, sand or snow. Values between 0.2 and 0.3 represent shrub and grassland, while higher values indicate denser green leaves (e.g. temperate and tropical rainforests) [42]. The variation in vegetation is derived from the standard deviation of NDVI for all green space within each neighbourhood. This indicator is influenced by the type of vegetation, such as trees, grass fields, shrub, woodland and forest. High levels of variation in vegetation characterize mixed vegetation within the neighbourhoods where both vegetation with high NDVI values (e.g. dense trees and woodland) and those with low NDVI values (e.g. grass field and shrub) exist together. Using NDVI as a measure of neighbourhood greenness has been validated against experts’ perception of greenness [43] and previously applied as a proxy for greenness in health and behaviour research [44-47].

(1) $$\text{NDVI}=\frac{\text{NearInfrared}-\text{Red}}{\text{NearInfrared}+\text{Red}}$$

**Figure 1:**
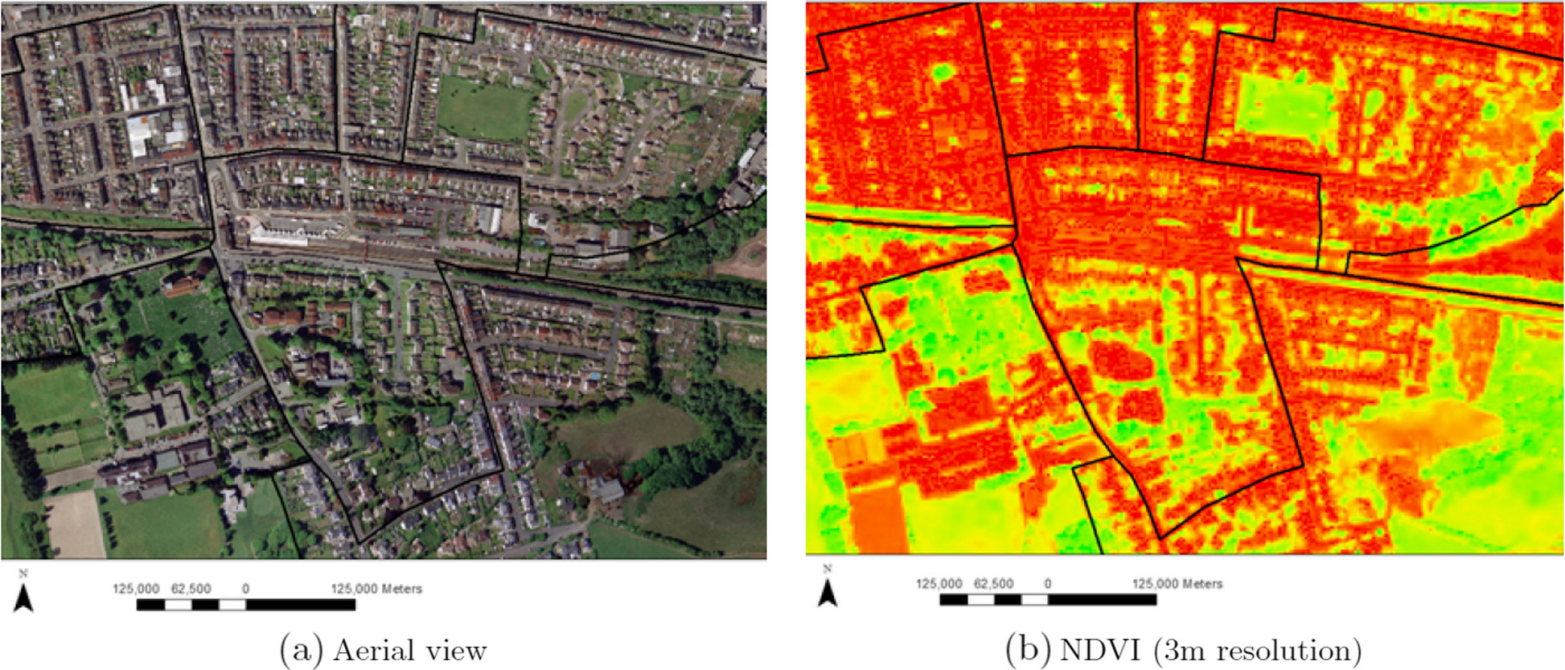
**An example of green space in Caerphilly county borough, Wales.(a)** Aerial view **(b)** NDVI (3m resolution).

**Figure 2:**
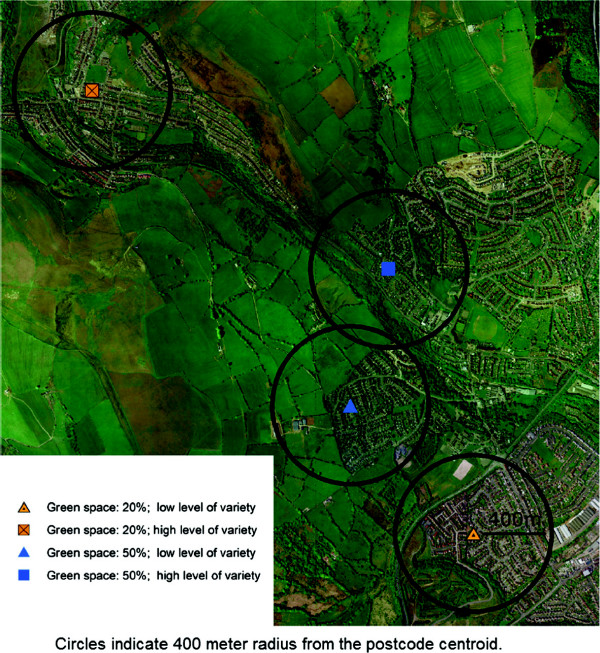
The amount of green space and the levels of variation in vegetation in four different neighbourhoods.

A high resolution (0.5 meter) true colour aerial photograph (Figure 1a) captured in 2006 was used to create a coarser 3-meter resolution NDVI for living vegetation for the study area, in order to optimise computation while still capturing small domestic gardens and other small green spaces (Figure 1b). The percentage of neighbourhood green space and variation in vegetation were calculated for each participant using a 400 meter buffer of a home address (geocoded as the centroid of a postcode). The variation in vegetation was then classified into two groups above and below median (high and low) and modelled as a two-level factor. Figure 2 shows four different neighbourhoods with different amounts (e.g. 20% and 50%) and levels of variation in vegetation (e.g. low and high) of green space within a 400 meter buffer of four different postcode centroids.

The area type is based on the rural and urban classification for census output areas 2004 by the Office for National Statistics [48]. This indicator is based on the population density: a census output area (OA) with over 10,000 people is defined as an urban area, while an output area with fewer than 10,000 people is a non-urban area. This variable contrasts residence in an urban area with residence in suburbs, small towns and rural areas. The area deprivation classification is based on the Townsend deprivation score from the 2001 Census [49], categorised into quintiles (highest, high, middle, low and lowest deprivation).

### Statistical analysis

The physical activity outcome was modelled as a binary variable using logistic regression where respondents who reported participation in any of three activities three or more times a week were characterized as regular participants in physical activity, whereas the others were characterised as irregular participants in physical activity.

Initially we tested the effect of neighbourhood green space (the quantity of green space, the variation in vegetation) adjusted for area type and area deprivation in Model 1. We modelled the amount of neighbourhood green space as a standardized z-score to make it easier to interpret the effect of neighbourhood green space on participation in physical activities. We then adjusted for lower extremity physical function, psychological distress, general health, car ownership, age group, marital status, social class and education level in Model 2. Finally we tested the modifying effect of lower extremity physical function by adding interaction terms (i.e. the amount of green space × lower extremity physical function, variation in vegetation × lower extremity physical function) to the full model fitted for the entire population. The p-value from the interaction term was used to determine if there was a statistically significant effect in physical activity participation between different levels of physical function over neighbourhood green space. If the interaction term reached statistical significance, we computed the predicted probabilities from the interaction model for subjects with poor and good lower extremity physical function, while holding covariates in the model constant at their means.

The missing socio-demographic and health status data was accounted for using multiple imputation by chained equations, including all individual variables in imputation models [50]. A total of five imputations were used to derive adjusted pooled odds ratios with 95% confidence intervals. All statistical analyses were performed using IBM PASW (version 18) and STATA (version 12).

## Results

Table 1 presents the socio-demographic characteristics of participants and their neighbourhood green space. The average age of the participants was 73.3 years (SD 4.09) and 39% were over 75 years old. 56% had CSE-level or higher education, 91% were married, 63% were manual social class and 73% owned at least one car. 52% of men reported good general health. 27% reported psychological distress and 52% poor lower extremity physical function. 53% of participants took part in physical activity regularly.

**Table 1:** Descriptive statistics for individuals and their neighbourhoods

					**Lower extremity physical function**			
	**All**				**Poor**		**Good**	
**Individual variables**	**N = 1010**	**%**			**N**	**%**	**N**	**%**
Age	1007				507		465	
67-74	616	61			297	56	299	44
75-85	391	39			210	50	166	50
Education	917				456		427	
No qualification	407	44			235	60	154	44
CSE and above	510	56			221	45	273	55
Marital status	967				486		446	
Single, windowed, divorced, separated	91	9			44	50	44	50
Married	879	91			442	52	402	48
Social class	965	60			484		446	
Non-manual	361	37			151	43	201	57
Manual	604	63			333	58	245	42
Car ownership	991				498		460	
No car	266	27			167	66	86	34
One or more cars	725	73			331	47	374	53
Psychological distress	857				430		399	
Yes	233	27			172	77	52	23
No	624	72			258	43	347	57
General health	999				505		460	
Poor	481	48			349	76	111	24
Good	518	52			156	31	349	69
Lower extremity physical function	975							
Poor	509	52						
Good	466	48						
**Area variables**	**N**	**%**			**Mean**	**SD**	**Mean**	**SD**
Area type	1010				509		466	
Non-urban area	205	20			99	49	102	51
Urban area	805	80			410	53	364	47
Area deprivation	1010				509		466	
Lowest deprivation	407	40			165	42	227	58
Lower deprivation	99	10			47	48	51	52
Middle deprivation	134	13			76	59	53	41
High deprivation	230	23			134	61	87	39
Highest deprivation	140	14			87	64	48	36
**Neighbourhood green space**	**N**	**%**	**Mean**	**SD**	**Mean**	**SD**	**Mean**	**SD**
Amount of green space (%)	1010		32%	0.12	31%	0.12	33%	0.13
Variation in vegetation	1010				509		466	
Low	505	50	0.10	0.02	0.10	0.02	0.10	0.02
High	505	50	0.15	0.02	0.15	0.02	0.15	0.02
**Outcome variable**	**N**	**%**			**N**	**%**	**N**	**%**
Frequency of physical activity participation	1010				509		466	
Irregular	472	47			300	66	155	34
Regular	538	53			209	40	311	60

The 1,010 participants lived in 486 neighbourhoods and 80% lived in urban areas. The amount of neighbourhood green space varied from 9.3% to 75.2%, with mean 32% (SD = 0.12). Descriptive statistics and group comparison of the variables for participants with good and poor lower extremity physical function showed that on average subjects with poor lower extremity physical function lived in less green neighbourhoods (Table 1).

### The association between physical activity and neighbourhood green space

A positive association between the amount of neighbourhood green space and regular participation in physical activity was observed in the unadjusted Model 1 (Table 2). Elderly men living in neighbourhoods with a higher amount of green space were significantly more likely to participate regularly in physical activity (OR = 1.25, 95% CI 1.09, 1.44). The odds ratio for regular physical activity was 0.88 (95% CI 0.67, 1.14) for those living in neighbourhoods with high variation in vegetation compared with those in low variation, and 0.46 (95% CI 0.31, 0.69) for those living in the highest deprived neighbourhoods compared with those in the least deprived.

**Table 2:** Odd Ratios (OR) for reported regular participation in physical activities

**N = 1010**	**Model 1**			**Model 2**		
	**OR**^**a**^	**95% CI**^**b**^	**P**	**OR**^**a**^	**95% CI**^**b**^	**P**
Amount of neighbourhood green space^c^ (%)	1.25	1.09, 1.44	0.002	1.21	1.05, 1.41	0.008
High variation in vegetation (vs. low)	0.88	0.67, 1.14	0.338	0.88	0.67, 1.16	0.375
Non-urban area (vs. urban area)	1.11	0.79, 1.56	0.553	1.01	0.76, 1.55	0.639
Townsend deprivation scores (vs. lowest deprivation)						
Lower deprivation	0.59	0.37, 0.93	0.023	0.64	0.40, 1.02	0.059
Middle deprivation	0.74	0.49, 1.13	0.166	0.92	0.59, 1.43	0.714
High deprivation	0.54	0.38, 0.76	<0.001	0.68	0.47, 0.99	0.042
Highest deprivation	0.46	0.31, 0.69	<0.001	0.63	0.41, 0.97	0.034
Good lower extremity physical function (vs. poor)				2.10	1.55, 2.85	<0.001
No psychological distress (vs. yes)				1.04	0.73, 1.47	0.844
Good general health (vs. poor)				1.41	1.04, 1.90	0.025
CSE-level and above education (vs. no)				0.98	0.72, 1.31	0.874
Own one or more car (vs. none)				1.59	1.15, 2.20	0.005
Age <=75 (vs. >75)				1.14	0.87, 1.50	0.353
Married (vs. single, window, divorced)				0.99	0.66, 1.50	0.969
Manual social class (vs. non-manual)				1.06	0.78, 1.44	0.705

^a^OR: Odds ratio, ^b^95% CI: 95% Confidence Interval, ^c^Modelled as a standardized z-score.

After adjusting for individual factors in Model 2 (Table 2), living in a neighbourhood with more green space remained significantly associated with regular participation in physical activity (OR = 1.21, 95% CI 1.05, 1.41). Equivalently, there was a 21% increase in the odds of regular participation in physical activity for a 1 standard deviation increase, or 12%, in the amount of green space. Subjects living in the highest deprived neighbourhoods were less likely to participate in regular physical activity than those from the least deprived neighbourhoods (OR = 0.63, 95% CI 0.41, 0.97). Good lower extremity physical function was associated with the largest effect on regular participation in physical activity (OR = 2.10, 95% CI 1.55, 2.85), followed by car ownership (OR = 1.59, 95% CI 1.15, 2.20) and good general health (OR = 1.41, 95% CI 1.04, 1.90). Table 3 shows the interaction between the amount of green space and lower extremity physical function was not statistically significant in Model 3 (OR = 0.92, 95% CI 0.70, 1.20).

**Table 3:** Odd Ratios for the interaction between the amount of neighbourhood green space and lower extremity physical function for reported regular participation in physical activities

**Model 3, N = 1010**^**a**^	**OR**^**b**^	**95% CI**^**c**^	**P**
Amount of neighbourhood green space^d^ (%)	1.27	1.04, 1.53	0.016
High variation in neighbourhood vegetation (vs. low)	0.88	0.67, 1.16	0.382
Good lower extremity physical function (vs. poor)	2.10	1.55, 2.84	<0.001
Interaction			
Amount of neighbourhood green space X good lower extremity physical function (vs. poor)	0.92	0.70, 1.20	0.530

^a^The analyses including the full study population were adjusted for the covariates: age, education, marital status, social class, car ownership, general health, psychological distress, urban/non-urban and area deprivation. ^b^OR: Odds Ratio, ^c^95% CI: 95% Confidence Interval, ^d^Modelled as a standardized z-score.

### Interaction between the variation in neighbourhood vegetation and lower extremity physical function

**Figure 3:**
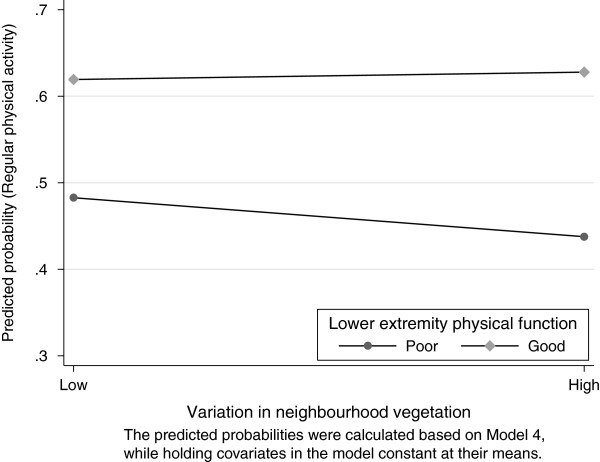
Predicted probability of regular participation in physical activities associated with variation in neighbourhood vegetation by the levels of lower extremity physical function.

In the interaction Model 4, the main effect for the variation in neighbourhood vegetation was now statistically significant (Table 4), such that living in a high variation vegetation neighbourhood was associated with a reduction in physical activity participation. The main effects for the amount of green space and good lower extremity physical function remained significantly associated with increased physical activity. Table 4 shows that the interaction between variation in neighbourhood vegetation and lower extremity physical function was statistically significant (p value = 0.017) in Model 4. Thus the association between the variation in neighbourhood vegetation and participation in regular physical activity varied with lower extremity physical function. Figure 3 shows the predicted probabilities of regular physical activity with increasing variation in neighbourhood vegetation for subjects with poor and good lower extremity physical function from Model 4, while holding covariates in the model constant at their means. Taking part in regular physical activity was significantly lower among those who reported poor lower extremity physical function in high vegetation variation neighbourhoods. Good lower extremity physical function significantly reduced the adverse effect on physical activity from living in a high variation neighbourhood.

**Table 4:** Odd Ratios for the interaction between variation in neighbourhood vegetation and lower extremity physical function for reported regular participation in physical activities

**Model 4, N = 1010**^**a**^	**OR**^**b**^	**95% CI**^**c**^	**P**
Amount of neighbourhood green space^d^ (%)	1.21	1.05, 1.40	0.009
High variation in neighbourhood vegetation (vs. low)	0.66	0.45, 0.95	0.025
Good lower extremity physical function (vs. poor)	1.52	1.02, 2.26	0.037
Interaction			
High variation in neighbourhood vegetation X good lower extremity physical function (vs. low variation and poor)	1.92	1.12, 3.28	0.017

^a^The analyses including the full study population were adjusted for the covariates: age, education, marital status, social class, car ownership, general health, psychological distress, urban/non-urban and area deprivation. ^b^OR: Odds Ratio, ^c^95% CI: 95% Confidence Interval, ^d^Modelled as a standardized z-score.

## Discussion

This study investigated the association between neighbourhood green space and participation in physical activity among elderly men with different levels of lower extremity physical function. Our first main finding is that the amount of neighbourhood green space was significantly associated with regular physical activity, regardless of lower extremity physical function level. The likelihood of regular physical activity was higher for those with good lower extremity physical function than for those with poor lower extremity physical function.

One possible underlying mechanism for this association is the opportunity that green space gives for increased physical activity. For example, people are more likely to walk, exercise and tend the garden in a greener environment. Similar observations were obtained among younger adults (aged 26-58 years) in England: people living in the greenest quintile Middle Layer Super Output Area (MSOA) were more likely to achieve the recommended amount of physical activity through different forms of physical activity, including gardening, DIY and occupational physical activity, compared to those living in the least green quintile [51].

Second, we observed a statistically significant interaction between variation in neighbourhood vegetation and lower extremity physical function, as they related to regular physical activity. Living in a neighbourhood with high variation in neighbourhood vegetation was significantly and negatively associated with participation in regular physical activity, but this adverse effect of high variation neighbourhoods was significantly reduced for subjects reporting good lower extremity physical function, compared to those reporting poor lower extremity physical function.

The positive and negative effect (for good and poor lower extremity physical function, respectively) of variation in neighbourhood vegetation on participation in physical activities was surprising. Low variation in neighbourhood vegetation suggests that most vegetation within a neighbourhood has a similar NDVI value. For example, the majority of vegetation would either have low NDVI values (e.g. grass field, shrub) or high NDVI values (e.g. dense trees, forests, and woodland). In contrast, high variation indicates that mixed vegetation (both high and low NDVI values) exists within the neighbourhood. It is possible that living in a neighbourhood with more homogeneous vegetation (i.e. low variation) is associated with fewer obstacles and challenges for those reporting poor lower extremity physical function to take part in physical activity, whereas high variation in vegetation might be more attractive for those reporting good lower extremity physical function.

Our results are consistent with the findings from three other studies which found that the effect of the neighbourhood environment on physical activity levels among older adults varies according to their level of lower extremity physical function [31-33]. If people are more likely to walk, exercise, garden and DIY in a greener environment, neighbourhood green space may have a role in supporting and maintaining active ageing for those individuals with limited physical function, as the provision of green space is modifiable through the planning process. This has implications for planning policy to design, preserve, facilitate and encourage the use of green space near home. More importantly, green space provision could have a more significant equity impact on those with different levels of lower extremity physical function. In addition, our results showed that relative to general physical and mental health, lower extremity physical function is an important factor associated with regular participation in physical activity. This is consistent with previous finding that better functional status was associated with greater physical activity in older age [52-57].

The strengths of this study include the use of a well-characterized established cohort with careful face-to-face measurement of all the variables. We used an objective measurement of neighbourhood green space at a fine spatial scale (3 meters). The green space in our analysis included both those formal and informal public green spaces (i.e. parks, playing fields, woods, road-side trees and roundabouts), and also those in private domains (i.e. private gardens). This is because the factor analysis of 22 activities suggested that gardening was in the same factor with the other physical activities and we recognised that gardening is an activity which could take place in a private space.

Limitations of this study include the cross-sectional study design which cannot establish causality. We used a self-reported frequency of physical activity and were limited by treating physical activity participation as a binary variable. All three activities have been treated equally although the intensity of activities may have varied from light-intensity at short duration to high-intensity at long duration. This could be further improved by applying pedometers and accelerometers to measure physical activity more accurately in future studies. The postcode centroids rather than the actual address were used to present participants’ home locations in the analysis, due to the availability of the data. The 400 meters radius buffer around postcode centroids might also not match exactly with the definitions of a neighbourhood as judged by an individual. Future studies could combine objective and perceived definitions of individual’s neighbourhood in their analyses. The study only focused on men and the associations might be different in women. There was a two-year mismatch on the data period of neighbourhood green space (2006) and the survey data (2004) due to green space data availability. However, it is highly unlikely that green space in Caerphilly changed significantly between those two years. First, the population of Caerphilly borough was estimated at 170,800 in mid-2004, only 300 fewer than in mid-2006 [58]. Such little variation suggested Caerphilly has been a relatively stable town characterized by low-density. Second, the studied area has not been subjected to any large-scale greening policy during those two years.

Finally, we lacked information on traffic and the quality of neighbourhood green space. It is possible that areas with less green space have more traffic, which may hinder older adults from participation in physical activity through visiting parks and open space. This needs to be investigated in future studies. Previous studies suggested that the quality of green space (e.g. aesthetic features) may play a role in the association [59], and people living in the most deprived areas in the UK are more likely to experience the poorest quality [60,61]. Arguably, we used urban/non-urban classification and area deprivation as a rather crude proxy for the quality of green space as with previous analyses and adjusted for these in the models.

## Conclusion

This study further contributes to the research evidence on the effects of neighbourhood green space on physical activity participation among elderly men with different levels of lower extremity physical function. Our results suggest that elderly men living in neighbourhoods with more green space participate in more regular physical activity. The association between variation in neighbourhood vegetation and regular physical activity varied according to physical function. Subjects reporting poor lower extremity physical function living in neighbourhoods with more homogeneous vegetation (i.e. low variation) were more likely to participate in regular physical activity than those living in neighbourhoods with less homogeneous vegetation (i.e. high variation). Good lower extremity physical function reduced the adverse effect of high variation in neighbourhood vegetation on participation in regular physical activity. Our work provides a basis for the future development of novel interventions that aim to support active ageing and increase levels of physical activity in later life. Policy makers should consider neighbourhood green space as a resource that supports elderly people’s participation in physical activity.

## Competing interests

The authors declare that they have no competing interests.

## Authors’ contributions

JG provided access to the Caerphilly Prospective study data. All authors made substantial contributions to the design, analysis and interpretation of data. All authors were involved in revising the manuscript and have approved the final manuscript.
